# Supplementary material for: Long COVID risk and pre-COVID vaccination in an EHR-based cohort study from the RECOVER program
Source: Nat Commun. 2023 May 22;14:2914. doi: 10.1038/s41467-023-38388-7 (PMC10201472; doi:10.1038/s41467-023-38388-7)
Supplement: Supplementary file 1 — Supplementary Information [file 41467_2023_38388_MOESM1_ESM.pdf]

Long COVID Risk and Pre-COVID Vaccination In An EHR-Based Cohort Study from the RECOVER Program

Supplementary Information

Supplementary Table 1. Number of Individuals with Each Long COVID Diagnosis in Clinic-based Cohort. Individuals can receive multiple diagnoses; some are included in multiple rows. The earliest diagnosis is used for proportional hazards modeling.

Long COVID Diagnosis	Count of Unique Individuals
U09.9 Diagnosis Code	661
B94.8 Diagnosis Code	79
Visit to a Long COVID Clinic	91

Supplementary Table 2. Distributions of Length of Follow-up for Both Cohorts. Length of follow-up is the time between COVID-19 infection and the end of a patient's follow-up period. The end of the follow-up period varies by contributing data partner.

Length of Follow-up (Days)	Model-Based Cohort (N=198514)	Clinic-Based Cohort (N=47404)
150-179	6821 (3.4)	2555 (5.4)
180-209	54329 (27.4)	18444 (38.9)
210-239	24590 (12.4)	5741 (12.1)
240-269	29931 (15.1)	2890 (6.1)
270-299	47362 (23.9)	3770 (8)
300-329	24471 (12.3)	7333 (15.5)
330-359	10757 (5.4)	6449 (13.6)
360-389	253 (0.1)	222 (0.5)

Supplementary Table 3. Model-based Logistic Regression, All Coefficients. The reference levels for categories with multiple indicators are: age, 35–49; race/ethnicity, white; data partner, J; COVID onset, August 2021.

Covariate	Coefficient	Standard Error	95% CI Low	95% CI High
Age: 18-24	-1.45	0.15	-1.75	-1.16
Age: 25-34	-0.80	0.08	-0.95	-0.65
Age: 50-64	0.17	0.05	0.07	0.27
Age: 65+	0.14	0.06	0.02	0.27
Acute Kidney Injury	0.23	0.10	0.03	0.43
Chronic Lung Disease	1.07	0.05	0.97	1.16
COVID In Sep 2021	-0.06	0.06	-0.18	0.05
COVID In Oct 2021	-0.23	0.07	-0.37	-0.09
COVID In Nov 2021	-0.18	0.07	-0.31	-0.05
COVID In Dec 2021	-0.33	0.07	-0.46	-0.19
COVID In Jan 2022	-0.78	0.07	-0.91	-0.64
Data Partner A	0.09	0.08	-0.07	0.25
Data Partner B	-0.19	0.22	-0.63	0.24
Data Partner C	-0.15	0.14	-0.43	0.13
Data Partner D	-0.82	0.24	-1.29	-0.35
Data Partner E	-0.56	0.24	-1.03	-0.09
Data Partner F	-0.51	0.07	-0.64	-0.37
Data Partner G	-0.03	0.13	-0.28	0.22
Data Partner H	0.01	0.13	-0.24	0.27
Data Partner I	0.92	0.13	0.66	1.18
Data Partner K	-1.53	0.06	-1.64	-1.42
Diabetes Complicated	0.01	0.09	-0.16	0.18
Diabetes Uncomplicated	0.24	0.07	0.11	0.38
Sex: Male	-0.21	0.05	-0.30	-0.11
Heart Failure	0.44	0.08	0.29	0.60
Immunocompromised	0.06	0.13	-0.20	0.32
Intercept	-3.06	0.07	-3.20	-2.93
Kidney Disease	0.00	0.08	-0.16	0.16
Myocardial Infarction	-0.08	0.10	-0.27	0.11
Race/Ethnicity: Asian	0.00	0.28	-0.55	0.56
Race/Ethnicity: Black	0.01	0.06	-0.10	0.13
Race/Ethnicity: Hispanic	-0.14	0.08	-0.30	0.02
Race/Ethnicity: Pacific Islander	0.32	0.25	-0.16	0.81
Race/Ethnicity: Other	-0.41	0.18	-0.77	-0.06
Race/Ethnicity: Unknown	-0.10	0.18	-0.46	0.26
Tobacco Smoker	-0.03	0.07	-0.17	0.10
Vaccination Status	-0.36	0.04	-0.44	-0.29

Supplementary Table 4. Clinic-based Logistic Regression, All Coefficients

Covariate	Coefficient	Standard Error	95% CI Low	95% CI High
Age: 18-24	-1.21	0.24	-1.67	-0.75
Age: 25-34	-0.63	0.17	-0.96	-0.30
Age: 50-64	0.14	0.11	-0.07	0.36
Age: 65+	0.24	0.12	0.00	0.49
Acute Kidney Injury	0.33	0.19	-0.03	0.70
Chronic Lung Disease	0.67	0.11	0.46	0.89
COVID In Sep 2021	-0.27	0.13	-0.53	0.00
COVID In Oct 2021	-0.41	0.16	-0.72	-0.10
COVID In Nov 2021	-0.24	0.17	-0.57	0.09
COVID In Dec 2021	-0.57	0.15	-0.87	-0.27
COVID In Jan 2022	-0.91	0.12	-1.16	-0.67
Data Partner A	-0.16	0.13	-0.42	0.10
Data Partner C	0.17	0.18	-0.18	0.51
Data Partner D	-0.14	0.26	-0.65	0.36
Data Partner G	0.34	0.12	0.10	0.58
Data Partner I	1.39	0.16	1.08	1.70
Diabetes Complicated	-0.10	0.21	-0.50	0.31
Diabetes Uncomplicated	0.08	0.16	-0.23	0.39
Sex: Male	-0.13	0.09	-0.31	0.05
Heart Failure	0.06	0.16	-0.24	0.37
Immunocompromised	-0.01	0.25	-0.49	0.47
Intercept	-3.72	0.12	-3.96	-3.48
Kidney Disease	0.18	0.15	-0.12	0.48
Myocardial Infarction	-0.48	0.23	-0.94	-0.03
Race/Ethnicity: Asian	0.38	0.31	-0.24	0.99
Race/Ethnicity: Black	-0.40	0.13	-0.66	-0.14
Race/Ethnicity: Hispanic	-0.11	0.16	-0.44	0.21
Race/Ethnicity: Other	0.06	0.27	-0.46	0.59
Race/Ethnicity: Unknown	0.07	0.27	-0.47	0.60
Tobacco Smoker	-0.22	0.15	-0.52	0.09
Vaccination Status	-0.36	0.08	-0.53	-0.20

Supplementary Table 5. Model-based Proportional Hazards, All Coefficients

Covariate	Coefficient	Standard Error	95% CI Low	95% CI High
Age: 18-24	-2.73	0.46	-3.63	-1.83
Age: 25-34	-0.89	0.10	-1.08	-0.71
Age: 50-64	0.27	0.06	0.15	0.39
Age: 65+	0.29	0.08	0.12	0.45
Acute Kidney Injury	0.13	0.10	-0.07	0.34
Chronic Lung Disease	1.01	0.06	0.88	1.14
COVID In Sep 2021	-0.07	0.07	-0.21	0.07
COVID In Oct 2021	-0.10	0.08	-0.27	0.07
COVID In Nov 2021	0.11	0.09	-0.05	0.28
COVID In Dec 2021	0.17	0.09	-0.02	0.35
COVID In Jan 2022	-0.22	0.12	-0.44	0.01
Data Partner A	0.31	0.10	0.11	0.51
Data Partner B	-0.36	0.26	-0.87	0.16
Data Partner C	-0.14	0.17	-0.47	0.19
Data Partner D	1.34	0.71	-0.06	2.74
Data Partner E	-0.38	0.35	-1.07	0.31
Data Partner F	-1.08	0.17	-1.43	-0.74
Data Partner G	-0.15	0.21	-0.57	0.26
Data Partner H	0.11	0.17	-0.22	0.44
Data Partner I	0.66	0.20	0.26	1.06
Data Partner K	-1.10	0.07	-1.24	-0.97
Diabetes Complicated	0.13	0.10	-0.07	0.33
Diabetes Uncomplicated	0.21	0.08	0.05	0.37
Sex: Male	0.25	0.12	0.01	0.49
Heart Failure	0.43	0.09	0.25	0.62
Immunocompromised	0.21	0.17	-0.13	0.54
Kidney Disease	-0.14	0.11	-0.36	0.08
Myocardial Infarction	0.00	0.13	-0.26	0.26
Race/Ethnicity: Asian	0.41	0.38	-0.32	1.15
Race/Ethnicity: Black	0.04	0.08	-0.11	0.19
Race/Ethnicity: Hispanic	-0.08	0.11	-0.29	0.13
Race/Ethnicity: Other	-0.30	0.22	-0.72	0.13
Race/Ethnicity: Unknown	-0.08	0.24	-0.56	0.39
Tobacco Smoker	0.03	0.09	-0.14	0.20
Vaccination Status	-0.46	0.05	-0.57	-0.36
Time * Age: 18-24	0.01	0.00	0.00	0.02
Time * Data Partner D	-0.02	0.01	-0.04	0.00
Time * Data Partner F	0.01	0.00	0.00	0.01
Time * Sex: Male	0.00	0.00	-0.01	0.00

Supplementary Table 6. Clinic-based Proportional Hazards, All Coefficients

Covariate	Coefficient	Standard Error	95% CI Low	95% CI High
Age: 18-24	-1.23	0.30	-1.82	-0.63
Age: 25-34	-0.54	0.19	-0.92	-0.17
Age: 50-64	0.27	0.13	0.01	0.53
Age: 65+	0.34	0.17	0.01	0.67
Acute Kidney Injury	0.40	0.21	-0.01	0.82
Chronic Lung Disease	0.70	0.12	0.46	0.94
COVID In Sep 2021	-0.08	0.14	-0.35	0.20
COVID In Oct 2021	-0.71	0.33	-1.36	-0.07
COVID In Nov 2021	-0.12	0.19	-0.51	0.26
COVID In Dec 2021	-0.40	0.19	-0.76	-0.03
COVID In Jan 2022	-0.53	0.17	-0.87	-0.20
Data Partner A	-0.02	0.15	-0.32	0.28
Data Partner C	0.12	0.21	-0.28	0.53
Data Partner D	-0.06	0.35	-0.74	0.62
Data Partner G	0.18	0.16	-0.14	0.50
Data Partner I	1.35	0.18	1.00	1.71
Diabetes Complicated	-0.26	0.28	-0.81	0.30
Diabetes Uncomplicated	0.07	0.18	-0.29	0.43
Sex: Male	-0.18	0.12	-0.41	0.06
Heart Failure	0.12	0.19	-0.26	0.50
Immunocompromised	-0.02	0.33	-0.66	0.62
Kidney Disease	0.18	0.17	-0.15	0.52
Myocardial Infarction	-0.52	0.27	-1.05	0.02
Race/Ethnicity: Asian	0.60	0.36	-0.11	1.32
Race/Ethnicity: Black	-0.38	0.14	-0.66	-0.11
Race/Ethnicity: Hispanic	-0.11	0.21	-0.52	0.30
Race/Ethnicity: Other	0.03	0.38	-0.71	0.76
Race/Ethnicity: Unknown	0.10	0.33	-0.55	0.75
Tobacco Smoker	-0.23	0.19	-0.61	0.14
Vaccination Status	-0.41	0.10	-0.60	-0.21
Time * COVID In Oct 2021	0.01	0.00	0.00	0.01
Time * Diabetes Complicated	0.00	0.00	0.00	0.01

Supplementary Table 7. Summary of Sensitivity Analyses. Cohort summaries and vaccine–long COVID associations for sensitivity analyses.

Sensitivity Definition	Cohort Size	Long COVID Prevalence	Logistic Regression OR[a] (95% CI)	Proportional Hazards HR[b] (95% CI)
≥89% Recorded Vaccine Ratio, Model-based	10,122	380 (3.8%)	0.66 (0.52, 0.84)	0.50 (0.34, 0.72)
≥89% Recorded Vaccine Ratio, Clinic-based	5,545	160 (2.9%)	0.62 (0.44, 0.88)	0.55 (0.36, 0.85)
Censor After Last Visit, Clinic-based	89,722	758 (0.8%)	-	0.66 (0.53, 0.80)
U09.9-only Diagnosis, Clinic-based	47,404	661 (1.4%)	0.69 (0.57, 0.83)	0.66 (0.54, 0.82)

Supplementary Table 8. Subanalysis Model-based Logistic Regression, All Coefficients. Coefficients from the subanalysis that includes indicators for length of time between an individual's most recent vaccine dose and acute COVID-19 onset.

Covariate	Coefficient	Standard Error	95% CI Low	95% CI High
Age: 18-24	-1.46	0.15	-1.74	-1.17
Age: 25-34	-0.80	0.07	-0.95	-0.65
Age: 50-64	0.17	0.05	0.06	0.27
Age: 65+	0.15	0.06	0.04	0.27
Acute Kidney Injury	0.23	0.09	0.05	0.40
Chronic Lung Disease	1.07	0.05	0.97	1.16
COVID In Sep 2021	-0.05	0.05	-0.15	0.06
COVID In Oct 2021	-0.21	0.07	-0.34	-0.07
COVID In Nov 2021	-0.15	0.08	-0.30	0.00
COVID In Dec 2021	-0.31	0.07	-0.45	-0.16
COVID In Jan 2022	-0.77	0.07	-0.91	-0.63
Data Partner A	0.09	0.09	-0.08	0.27
Data Partner B	-0.20	0.21	-0.61	0.22
Data Partner C	-0.15	0.13	-0.42	0.11
Data Partner D	-0.83	0.23	-1.27	-0.38
Data Partner E	-0.57	0.23	-1.02	-0.12
Data Partner F	-0.51	0.06	-0.63	-0.38
Data Partner G	-0.03	0.11	-0.25	0.19
Data Partner H	0.01	0.13	-0.24	0.26
Data Partner I	0.92	0.14	0.65	1.19
Data Partner K	-1.53	0.06	-1.65	-1.42
Diabetes Complicated	0.01	0.08	-0.16	0.17
Diabetes Uncomplicated	0.24	0.06	0.12	0.37
Sex: Male	-0.21	0.05	-0.30	-0.12
Heart Failure	0.44	0.08	0.30	0.59
Immunocompromised	0.04	0.15	-0.24	0.33
Intercept	-3.08	0.07	-3.22	-2.93
Kidney Disease	0.00	0.08	-0.16	0.15
Myocardial Infarction	-0.08	0.10	-0.28	0.11
Race/Ethnicity: Asian	0.00	0.28	-0.54	0.54
Race/Ethnicity: Black	0.01	0.06	-0.10	0.12
Race/Ethnicity: Hispanic	-0.14	0.09	-0.32	0.04
Race/Ethnicity: Pacific Islander	0.33	0.26	-0.19	0.84
Race/Ethnicity: Other	-0.41	0.16	-0.73	-0.10
Race/Ethnicity: Unknown	-0.10	0.19	-0.46	0.27
Tobacco Smoker	-0.04	0.07	-0.18	0.10
Vaccination Status	-0.45	0.05	-0.54	-0.35
Weeks since last vaccine: <2	0.12	0.16	-0.20	0.45
Weeks since last vaccine: 2-4	0.38	0.16	0.06	0.70
Weeks since last vaccine: 5-9	0.02	0.11	-0.20	0.24
Weeks since last vaccine: 10-14	0.21	0.12	-0.02	0.43
Weeks since last vaccine: 15-19	0.26	0.09	0.08	0.45
Weeks since last vaccine: 20-24	0.09	0.09	-0.08	0.26

Supplementary Table 9. Subanalysis Clinic-based Logistic Regression, All Coefficients. Coefficients from the subanalysis that includes indicators for length of time between an individual's most recent vaccine dose and acute COVID-19 onset.

Covariate	Coefficient	Standard Error	95% CI Low	95% CI High
Age: 18-24	-1.21	0.24	-1.69	-0.74
Age: 25-34	-0.63	0.16	-0.95	-0.30
Age: 50-64	0.14	0.11	-0.08	0.36
Age: 65+	0.24	0.14	-0.03	0.50
Acute Kidney Injury	0.33	0.19	-0.03	0.70
Chronic Lung Disease	0.67	0.10	0.47	0.87
COVID In Sep 2021	-0.26	0.13	-0.52	-0.01
COVID In Oct 2021	-0.39	0.17	-0.72	-0.06
COVID In Nov 2021	-0.22	0.17	-0.56	0.12
COVID In Dec 2021	-0.54	0.16	-0.86	-0.21
COVID In Jan 2022	-0.88	0.13	-1.14	-0.63
Data Partner A	-0.16	0.13	-0.42	0.10
Data Partner C	0.17	0.19	-0.20	0.53
Data Partner D	-0.14	0.28	-0.68	0.41
Data Partner G	0.34	0.13	0.08	0.60
Data Partner I	1.39	0.15	1.09	1.69
Diabetes Complicated	-0.10	0.19	-0.47	0.27
Diabetes Uncomplicated	0.08	0.14	-0.20	0.36
Sex: Male	-0.13	0.09	-0.30	0.03
Heart Failure	0.06	0.18	-0.29	0.41
Immunocompromised	-0.01	0.23	-0.47	0.45
Intercept	-3.73	0.13	-3.99	-3.47
Kidney Disease	0.18	0.15	-0.12	0.48
Myocardial Infarction	-0.48	0.21	-0.90	-0.06
Race/Ethnicity: Asian	0.39	0.30	-0.19	0.97
Race/Ethnicity: Black	-0.40	0.14	-0.68	-0.13
Race/Ethnicity: Hispanic	-0.12	0.19	-0.49	0.26
Race/Ethnicity: Other	0.06	0.28	-0.48	0.61
Race/Ethnicity: Unknown	0.07	0.25	-0.42	0.56
Tobacco Smoker	-0.21	0.17	-0.54	0.11
Vaccination Status	-0.37	0.10	-0.58	-0.17
Weeks since last vaccine: <2	0.28	0.29	-0.29	0.84
Weeks since last vaccine: 2-4	0.19	0.21	-0.22	0.60
Weeks since last vaccine: 5-9	-0.56	0.28	-1.10	-0.01
Weeks since last vaccine: 10-14	0.00	0.23	-0.45	0.45
Weeks since last vaccine: 15-19	-0.03	0.22	-0.46	0.40
Weeks since last vaccine: 20-24	0.17	0.18	-0.19	0.53

Supplementary Table 10. Subanalysis Model-based Proportional Hazards, All Coefficients. Coefficients from the subanalysis that includes indicators for length of time between an individual's most recent vaccine dose and acute COVID-19 onset.

Covariate	Coefficient	Standard Error	95% CI Low	95% CI High
Age: 18-24	-2.73	0.46	-3.63	-1.82
Age: 25-34	-0.90	0.10	-1.09	-0.70
Age: 50-64	0.27	0.07	0.14	0.40
Age: 65+	0.29	0.08	0.14	0.45
Acute Kidney Injury	0.13	0.13	-0.12	0.38
Chronic Lung Disease	1.01	0.07	0.87	1.14
COVID In Sep 2021	-0.07	0.07	-0.21	0.07
COVID In Oct 2021	-0.09	0.09	-0.26	0.08
COVID In Nov 2021	0.13	0.10	-0.06	0.32
COVID In Dec 2021	0.18	0.11	-0.03	0.38
COVID In Jan 2022	-0.21	0.10	-0.40	-0.02
Data Partner A	0.31	0.10	0.10	0.51
Data Partner B	-0.36	0.25	-0.86	0.14
Data Partner C	-0.14	0.17	-0.47	0.19
Data Partner D	1.34	2.41	-3.38	6.06
Data Partner E	-0.39	0.35	-1.07	0.29
Data Partner F	-1.09	0.20	-1.47	-0.70
Data Partner G	-0.16	0.20	-0.55	0.24
Data Partner H	0.11	0.16	-0.20	0.42
Data Partner I	0.66	0.22	0.22	1.10
Data Partner K	-1.11	0.07	-1.24	-0.97
Diabetes Complicated	0.13	0.12	-0.10	0.37
Diabetes Uncomplicated	0.21	0.09	0.03	0.38
Sex: Male	0.25	0.14	-0.02	0.52
Heart Failure	0.43	0.10	0.24	0.63
Immunocompromised	0.20	0.18	-0.15	0.55
Kidney Disease	-0.14	0.11	-0.36	0.07
Myocardial Infarction	0.00	0.13	-0.25	0.25
Race/Ethnicity: Asian	0.41	0.41	-0.39	1.22
Race/Ethnicity: Black	0.04	0.08	-0.12	0.19
Race/Ethnicity: Hispanic	-0.08	0.12	-0.32	0.15
Race/Ethnicity: Other	-0.30	0.20	-0.69	0.09
Race/Ethnicity: Unknown	-0.08	0.25	-0.57	0.40
Tobacco Smoker	0.03	0.09	-0.15	0.20
Vaccination Status	-0.54	0.07	-0.68	-0.40
Weeks since last vaccine: <2	0.03	0.22	-0.41	0.47
Weeks since last vaccine: 2-4	0.39	0.21	-0.01	0.79
Weeks since last vaccine: 5-9	0.07	0.19	-0.30	0.44
Weeks since last vaccine: 10-14	0.16	0.16	-0.15	0.47
Weeks since last vaccine: 15-19	0.17	0.14	-0.11	0.45
Weeks since last vaccine: 20-24	0.08	0.14	-0.19	0.36
Time * Age: 18-24	0.01	0.00	0.00	0.02
Time * Data Partner D	-0.02	0.04	-0.11	0.06
Time * Data Partner F	0.01	0.00	0.00	0.01
Time * Sex: Male	0.00	0.00	-0.01	0.00

Supplementary Table 11. Subanalysis Clinic-based Proportional Hazards, All Coefficients. Coefficients from the subanalysis that includes indicators for length of time between an individual's most recent vaccine dose and acute COVID-19 onset.

Covariate	Coefficient	Standard Error	95% CI Low	95% CI High
Age: 18-24	-1.23	0.31	-1.84	-0.62
Age: 25-34	-0.55	0.19	-0.92	-0.17
Age: 50-64	0.27	0.14	-0.02	0.55
Age: 65+	0.34	0.17	0.01	0.67
Acute Kidney Injury	0.41	0.22	-0.02	0.83
Chronic Lung Disease	0.70	0.12	0.46	0.93
COVID In Sep 2021	-0.07	0.14	-0.34	0.19
COVID In Oct 2021	-0.34	0.19	-0.72	0.04
COVID In Nov 2021	-0.11	0.22	-0.53	0.32
COVID In Dec 2021	-0.37	0.21	-0.77	0.04
COVID In Jan 2022	-0.51	0.15	-0.81	-0.20
Data Partner A	-0.02	0.15	-0.31	0.27
Data Partner C	0.12	0.22	-0.30	0.55
Data Partner D	-0.06	0.30	-0.64	0.52
Data Partner G	0.18	0.19	-0.19	0.54
Data Partner I	1.35	0.18	1.00	1.71
Diabetes Complicated	-0.11	0.21	-0.53	0.31
Diabetes Uncomplicated	0.07	0.17	-0.26	0.40
Sex: Male	-0.18	0.11	-0.39	0.03
Heart Failure	0.12	0.20	-0.28	0.52
Immunocompromised	-0.01	0.30	-0.61	0.58
Kidney Disease	0.18	0.16	-0.14	0.50
Myocardial Infarction	-0.52	0.28	-1.06	0.03
Race/Ethnicity: Asian	0.61	0.40	-0.18	1.40
Race/Ethnicity: Black	-0.38	0.15	-0.68	-0.08
Race/Ethnicity: Hispanic	-0.11	0.23	-0.55	0.33
Race/Ethnicity: Other	0.03	0.35	-0.65	0.71
Race/Ethnicity: Unknown	0.10	0.33	-0.54	0.74
Tobacco Smoker	-0.23	0.17	-0.57	0.10
Vaccination Status	-0.42	0.13	-0.67	-0.17
Weeks since last vaccine: <2	0.27	0.44	-0.60	1.14
Weeks since last vaccine: 5-9	-0.43	0.36	-1.12	0.27
Weeks since last vaccine: 10-14	0.11	0.25	-0.39	0.60
Weeks since last vaccine: 15-19	0.04	0.25	-0.44	0.52
Weeks since last vaccine: 20-24	0.07	0.24	-0.41	0.55

Supplementary Figure 1. Differences in standardized covariates in the model-based cohort. Comparing vaccinated and unvaccinated groups before (unadjusted) and after (adjusted) IPTW.

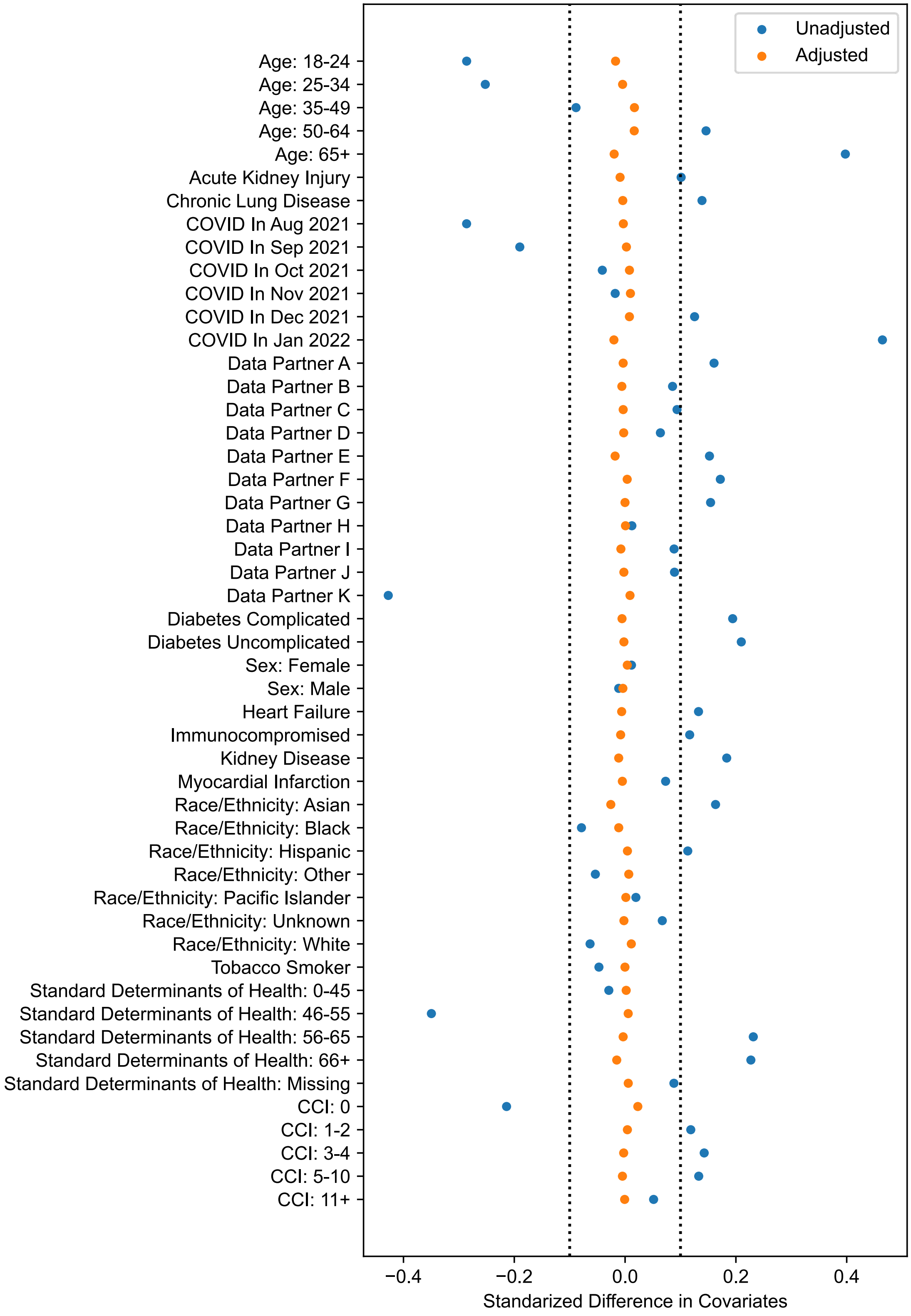

Supplementary Figure 2. Differences in standardized covariates in the clinic-based cohort. Comparing vaccinated and unvaccinated groups before (unadjusted) and after (adjusted) IPTW.

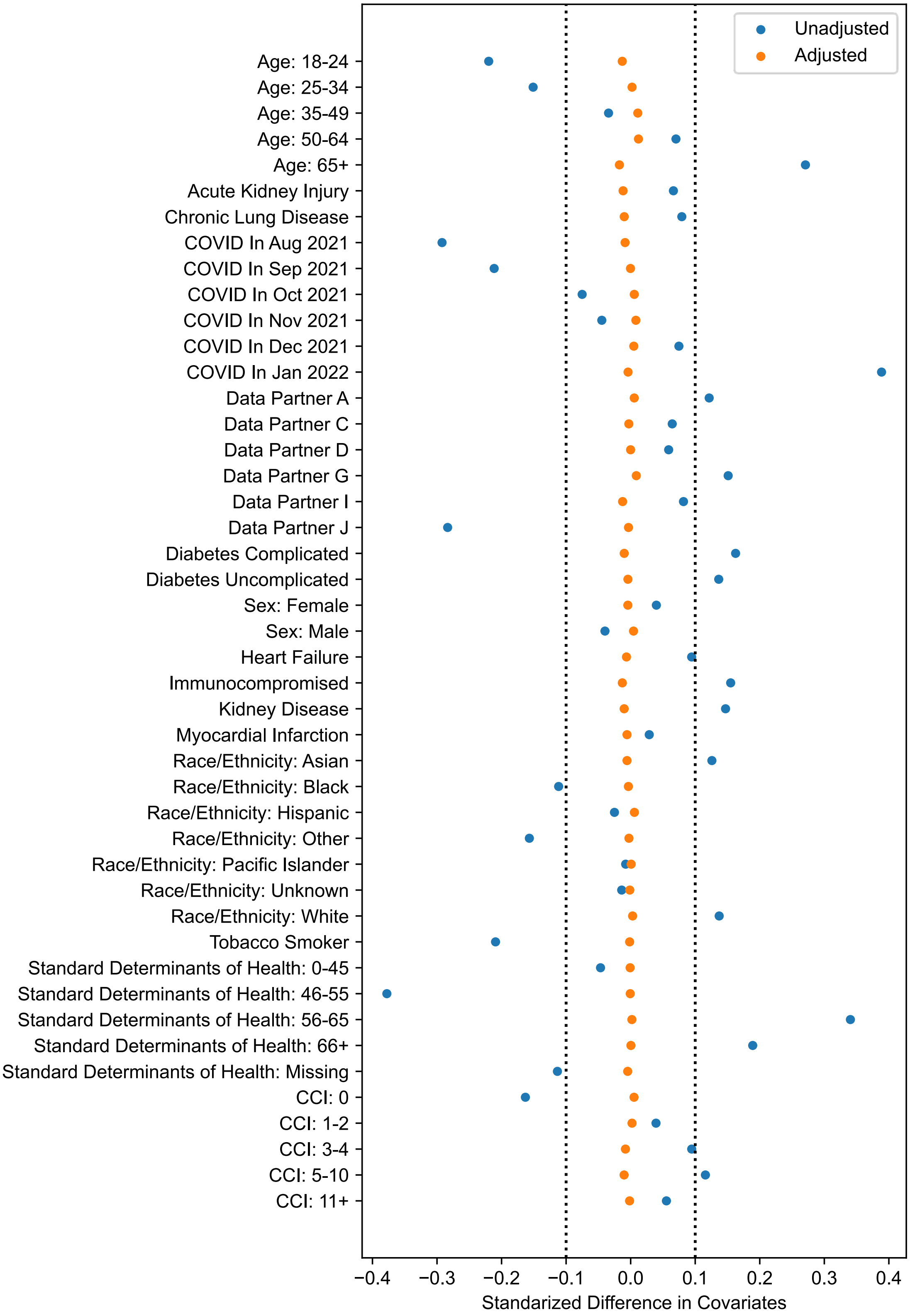

Supplementary Table 12. Model-Based IPTW Model Coefficients

Covariate	Coefficient	Standard Error	95% CI Low	95% CI High
Age: 18-24	-0.80	0.02	-0.84	-0.76
Age: 25-34	-0.44	0.02	-0.47	-0.41
Age: 50-64	0.44	0.01	0.41	0.47
Age: 65+	1.09	0.02	1.06	1.12
Acute Kidney Injury	-0.23	0.03	-0.29	-0.18
CCI: 1-2	0.04	0.01	0.01	0.07
CCI: 3-4	0.10	0.02	0.06	0.15
CCI: 5-10	0.14	0.03	0.08	0.21
CCI: 11+	0.16	0.09	-0.01	0.33
Chronic Lung Disease	0.03	0.02	0.00	0.07
COVID In Sep 2021	0.13	0.02	0.10	0.15
COVID In Oct 2021	0.33	0.02	0.30	0.36
COVID In Nov 2021	0.42	0.02	0.39	0.46
COVID In Dec 2021	0.72	0.02	0.68	0.75
COVID In Jan 2022	1.31	0.02	1.27	1.35
Data Partner A	0.40	0.03	0.34	0.45
Data Partner B	0.28	0.06	0.17	0.39
Data Partner C	0.37	0.04	0.29	0.45
Data Partner D	0.77	0.07	0.64	0.91
Data Partner E	0.44	0.06	0.34	0.55
Data Partner F	0.08	0.03	0.03	0.12
Data Partner G	0.75	0.03	0.69	0.81
Data Partner H	-0.09	0.05	-0.19	0.00
Data Partner I	0.62	0.05	0.52	0.72
Data Partner K	-0.07	0.02	-0.10	-0.04
Diabetes Complicated	0.13	0.03	0.08	0.18
Diabetes Uncomplicated	0.06	0.02	0.02	0.09
Sex: Male	-0.14	0.01	-0.16	-0.12
Heart Failure	-0.01	0.03	-0.07	0.05
Immunocompromised	0.58	0.05	0.47	0.69
Intercept	-1.04	0.02	-1.08	-1.00
Kidney Disease	0.17	0.02	0.12	0.22
Myocardial Infarction	-0.15	0.03	-0.22	-0.09
Race/Ethnicity: Asian	0.82	0.05	0.72	0.93
Race/Ethnicity: Black	-0.48	0.02	-0.51	-0.45
Race/Ethnicity: Hispanic	0.14	0.02	0.10	0.17
Race/Ethnicity: Pacific Islander	-0.23	0.14	-0.50	0.04
Race/Ethnicity: Other	-0.37	0.04	-0.44	-0.30
Race/Ethnicity: Unknown	0.28	0.04	0.20	0.35
SDOH: 0-45	-0.31	0.07	-0.46	-0.17
SDOH: 56-65	0.54	0.01	0.52	0.57
SDOH: 65+	1.05	0.03	0.99	1.12
SDOH: Missing	0.14	0.03	0.09	0.19
Tobacco Smoker	-0.56	0.03	-0.61	-0.51

Supplementary Table 13. Clinic-Based IPTW Model Coefficients

Covariate	Coefficient	Standard Error	95% CI Low	95% CI High
Age: 18-24	-0.76	0.04	-0.84	-0.68
Age: 25-34	-0.34	0.03	-0.40	-0.28
Age: 50-64	0.23	0.03	0.18	0.29
Age: 65+	0.69	0.03	0.63	0.76
Acute Kidney Injury	-0.30	0.05	-0.39	-0.20
CCI: 1-2	0.08	0.03	0.03	0.14
CCI: 3-4	0.16	0.04	0.07	0.24
CCI: 5-10	0.23	0.05	0.13	0.34
CCI: 11+	0.27	0.12	0.03	0.51
Chronic Lung Disease	0.06	0.03	0.01	0.12
COVID In Sep 2021	0.17	0.04	0.10	0.24
COVID In Oct 2021	0.34	0.05	0.26	0.43
COVID In Nov 2021	0.40	0.05	0.30	0.49
COVID In Dec 2021	0.90	0.04	0.83	0.97
COVID In Jan 2022	1.30	0.03	1.24	1.36
Data Partner A	0.28	0.03	0.23	0.34
Data Partner C	0.28	0.04	0.20	0.36
Data Partner D	0.63	0.07	0.49	0.76
Data Partner G	0.61	0.03	0.55	0.68
Data Partner I	0.62	0.05	0.51	0.72
Diabetes Complicated	0.24	0.05	0.14	0.33
Diabetes Uncomplicated	0.05	0.04	-0.02	0.12
Sex: Male	-0.19	0.02	-0.23	-0.14
Heart Failure	-0.01	0.05	-0.11	0.09
Immunocompromised	0.82	0.08	0.67	0.97
Intercept	-0.97	0.04	-1.04	-0.90
Kidney Disease	0.16	0.04	0.07	0.24
Myocardial Infarction	-0.14	0.05	-0.24	-0.03
Race/Ethnicity: Asian	0.67	0.09	0.50	0.84
Race/Ethnicity: Black	-0.37	0.03	-0.42	-0.32
Race/Ethnicity: Hispanic	-0.24	0.04	-0.32	-0.16
Race/Ethnicity: Pacific Islander	-0.55	0.26	-1.05	-0.04
Race/Ethnicity: Other	-0.73	0.06	-0.85	-0.60
Race/Ethnicity: Unknown	-0.23	0.07	-0.36	-0.10
SDOH: 0-45	-0.50	0.18	-0.86	-0.14
SDOH: 56-65	0.70	0.02	0.65	0.74
SDOH: 65+	1.34	0.06	1.23	1.45
SDOH: Missing	-0.38	0.08	-0.53	-0.23
Tobacco Smoker	-0.66	0.03	-0.72	-0.59

Supplementary Table 14. Associations of Age with Vaccination and Age with Long COVID

	Age at COVID Index Date	Overall	Fully Vaccinated	Unvaccinated	With Long COVID	Without Long COVID
Model-based Cohort	All	198514 (100.0)	86248 (100.0)	112266 (100.0)	3391 (100.0)	195123 (100.0)
	18-24	20603 (10.4)	4841 (5.6)	15762 (14.0)	71 (2.1)	20532 (10.5)
	25-34	36515 (18.4)	11197 (13.0)	25318 (22.6)	292 (8.6)	36223 (18.6)
	35-49	53548 (27.0)	21355 (24.8)	32193 (28.7)	978 (28.8)	52570 (26.9)
	50-64	50644 (25.5)	25118 (29.1)	25526 (22.7)	1169 (34.5)	49475 (25.4)
	65+	37204 (18.7)	23737 (27.5)	13467 (12.0)	881 (26.0)	36323 (18.6)
Clinic-based Cohort	All	47404 (100.0)	26354 (100.0	21050 (100.0)	695 (100.0)	46709 (100.0)
	18-24	4483 (9.5)	1731 (6.6)	2752 (13.1)	21 (3.0)	4462 (9.6)
	25-34	8486 (17.9)	4037 (15.3)	4449 (21.1)	67 (9.6)	8419 (18.0)
	35-49	12334 (26.0)	6681 (25.4)	5653 (26.9)	193 (27.8)	12141 (26.0)
	50-64	12268 (25.9)	7179 (27.2)	5089 (24.2)	227 (32.7)	12041 (25.8)
	65+	9833 (20.7)	6726 (25.5)	3107 (14.8)	187 (26.9)	9646 (20.7)

Supplementary Figure 3. Sensitivity Analysis Results for Full Threshold Range. Odds ratios for the vaccination coefficient are shown for logistic regression (LR), hazard ratios for the vaccination coefficient are shown for proportional hazards (PH). Dashed lines are results for the clinic-based outcome, where the computational phenotype score cutoff is not relevant. Solid lines are results for the model-based outcome.

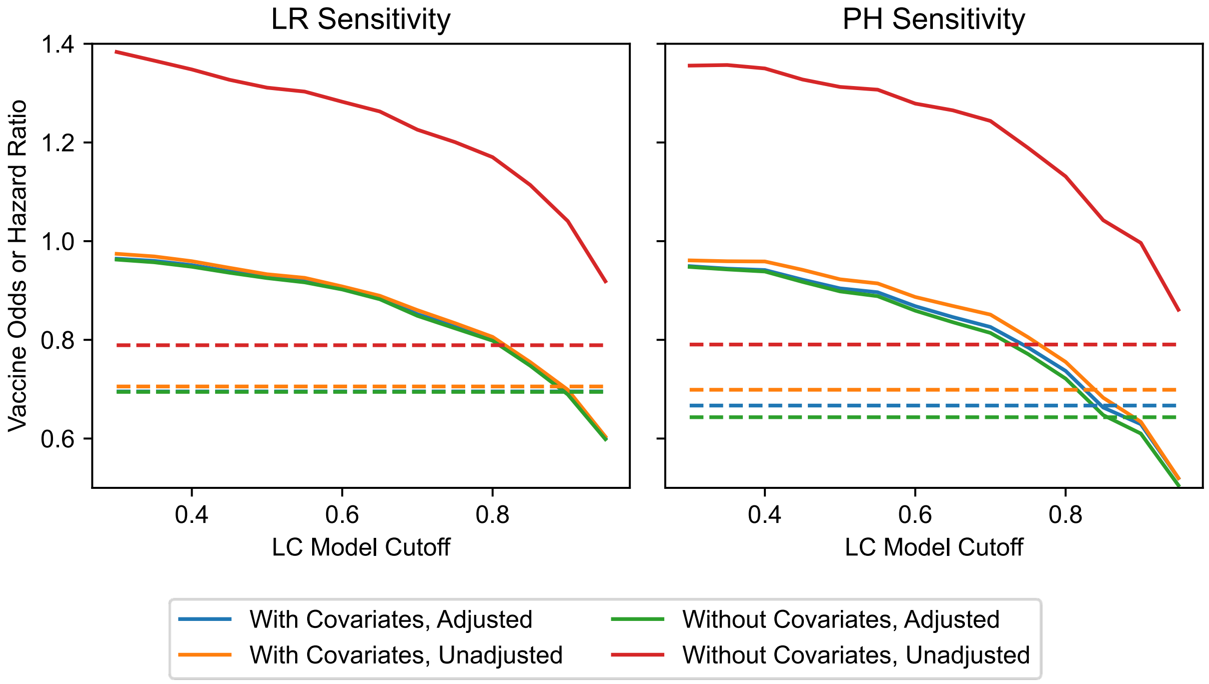

Supplementary Discussion

We do not interpret the associations between vaccination and long COVID here as causal, as we fail to fully account for two important conditions: unconfoundedness and latent variables.

Unconfoundedness

Under certain assumptions, associations in IPTW-adjusted models can be interpreted as causal effects, even when treatment is not randomly assigned.[1,2] If we are willing to assume that there are no unmeasured confounders, IPTW satisfies the condition of exchangeability: the treatment and control groups differ in outcome only due to the treatment. We attempt to satisfy this assumption by controlling for the measured confounders outlined in Supplementary Tables 1 and 2 in our treatment model. Nevertheless, we do not assume that there are no further confounders, largely due to the field's nascent understanding of long COVID. For example, a small cohort study suggested that reactivation of latent viruses may contribute to long COVID, but we do not include past viral infections in the treatment model.[3] Furthermore, some of the latent variables in our causal model are unobserved confounders, as discussed further below.

Latent Variables

To assess the feasibility of interpreting our results causally, we developed a simplified, theoretical causal model of COVID-19 and long COVID. This model is illustrated with a directed acyclic graph (DAG) in Supplementary Figure 4 and reveals latent variables that present an obstacle to estimating the causal effect of COVID-19 vaccination on long COVID. Of particular concern is patient propensity to seek healthcare, which affects the likelihood that both COVID-19 vaccination and long COVID will be observed, and is common in research using electronic health records.[4]

Supplementary Figure 4: Directed Acyclic Graph of COVID-19 Vaccination and Long COVID. This simplified, theoretical causal model graph illustrates our hesitancy in assigning causality to the association between vaccination and long COVID. There are several latent factors that may influence both the treatment and outcome that cannot be accounted for in the EHR.

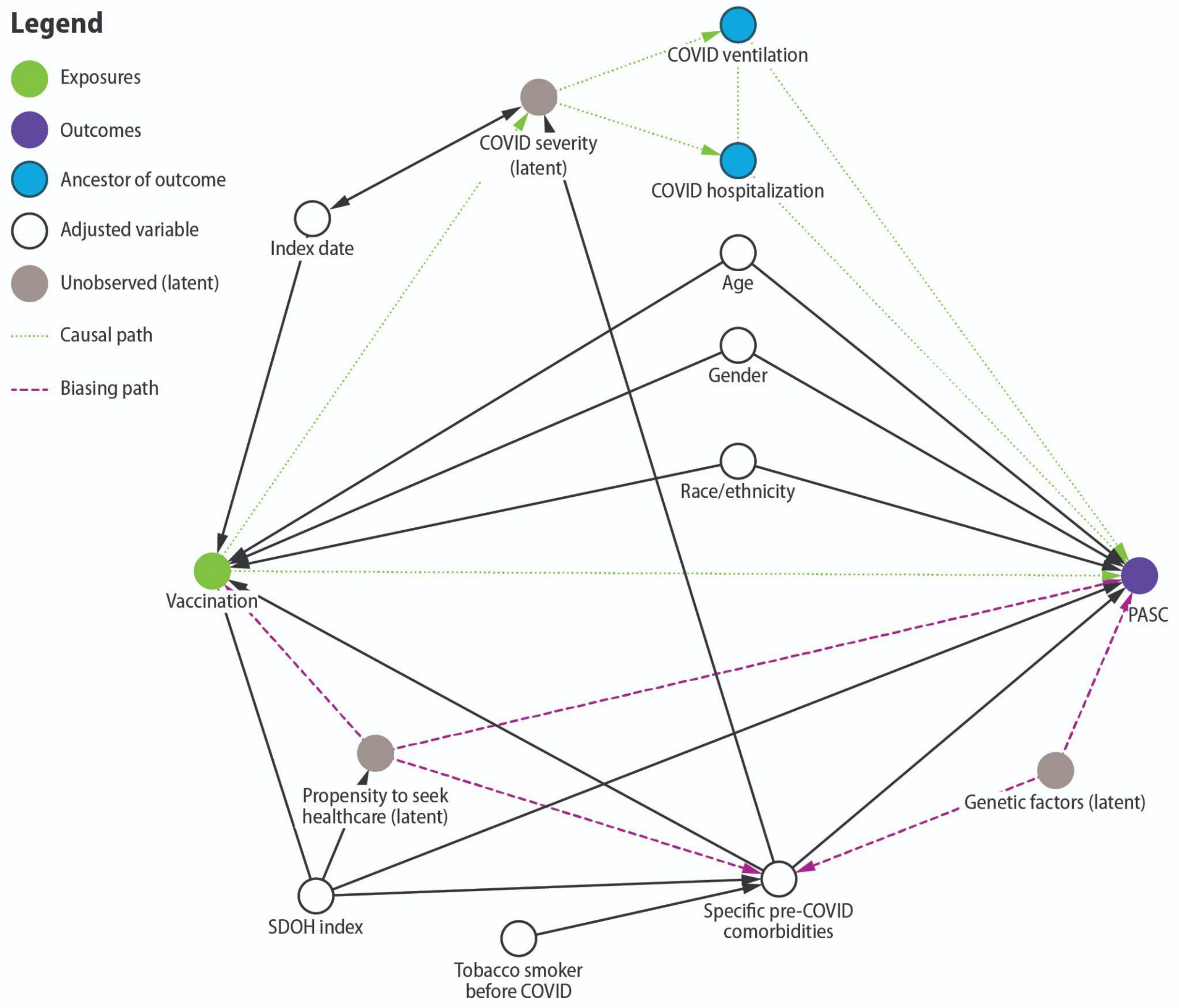

Supplemental References

1. Hernán MA, Robins JM. Estimating causal effects from epidemiological data. *J Epidemiol Community Health*. 2006;60(7):578-586.

2. Chesnaye NC, Stel VS, Tripepi G, et al. An introduction to inverse probability of treatment weighting in observational research. *Clin Kidney J*. 2022;15(1):14-20.

3. Su Y, Yuan D, Chen DG, et al. Multiple early factors anticipate post-acute COVID-19 sequelae. *Cell*. 2022;185(5):881-895.e20.

4. Farmer R, Mathur R, Bhaskaran K, Eastwood SV, Chaturvedi N, Smeeth L. Promises and pitfalls of electronic health record analysis. *Diabetologia*. 2018;61(6):1241-1248.
