# Supplementary material for: Long COVID risk and pre-COVID vaccination in an EHR-based cohort study from the RECOVER program
Source: Nat Commun. 2023 May 22;14:2914. doi: 10.1038/s41467-023-38388-7 (PMC10201472; doi:10.1038/s41467-023-38388-7)
Supplement: Supplementary file 3 — Reporting Summary [file 41467_2023_38388_MOESM3_ESM.pdf]

nature portfolio

Corresponding author(s): M Daniel Brannock

Last updated by author(s): Apr 20, 2023

Reporting Summary

Nature Portfolio wishes to improve the reproducibility of the work that we publish. This form provides structure for consistency and transparency in reporting. For further information on Nature Portfolio policies, see our Editorial Policies and the Editorial Policy Checklist.

Statistics

For all statistical analyses, confirm that the following items are present in the figure legend, table legend, main text, or Methods section.

n/a	Confirmed	
☐	☒	The exact sample size (n) for each experimental group/condition, given as a discrete number and unit of measurement
☒	☐	A statement on whether measurements were taken from distinct samples or whether the same sample was measured repeatedly
☒	☐	The statistical test(s) used AND whether they are one- or two-sided<br>*Only common tests should be described solely by name; describe more complex techniques in the Methods section.*
☐	☒	A description of all covariates tested
☐	☒	A description of any assumptions or corrections, such as tests of normality and adjustment for multiple comparisons
☐	☒	A full description of the statistical parameters including central tendency (e.g. means) or other basic estimates (e.g. regression coefficient) AND variation (e.g. standard deviation) or associated estimates of uncertainty (e.g. confidence intervals)
☒	☐	For null hypothesis testing, the test statistic (e.g. F, t, r) with confidence intervals, effect sizes, degrees of freedom and P value noted<br>*Give P values as exact values whenever suitable.*
☒	☐	For Bayesian analysis, information on the choice of priors and Markov chain Monte Carlo settings
☒	☐	For hierarchical and complex designs, identification of the appropriate level for tests and full reporting of outcomes
☒	☐	Estimates of effect sizes (e.g. Cohen's d, Pearson's r), indicating how they were calculated

Our web collection on statistics for biologists contains articles on many of the points above.

Software and code

Policy information about availability of computer code

Data collection	Data is contributed to the National COVID Cohort Collaborative (N3C) by 76 partner hospitals and hospital networks, each with their own suite of software that includes R, Python, TriNetX, and SQL. The specific software used to submit data to N3C is unique to each partner and not available to researchers. How data is collected in N3C has been previously described (https://academic.oup.com/jamia/article/28/3/427/5893482). Data was combined for analysis with Python (v3.6.10), PySpark (v3.2.1), pandas (v0.25.3), numpy (v1.19.5), and R (v3.5.1).
Data analysis	Python (v3.6.10) was used with the Statsmodels (v0.12.2) and Lifelines (v0.26.4) libraries for analysis. Data was prepared for analysis using Python (v3.6.10) with the pyspark (v3.2.1), pandas (v0.25.3), and numpy (v1.19.5) libraries.

For manuscripts utilizing custom algorithms or software that are central to the research but not yet described in published literature, software must be made available to editors and reviewers. We strongly encourage code deposition in a community repository (e.g. GitHub). See the Nature Portfolio guidelines for submitting code & software for further information.

Data

Policy information about availability of data

All manuscripts must include a data availability statement. This statement should provide the following information, where applicable:

- Accession codes, unique identifiers, or web links for publicly available datasets
- A description of any restrictions on data availability
- For clinical datasets or third party data, please ensure that the statement adheres to our policy

The data for this study was collected as a part of the National COVID Cohort Collaborative (N3C), which then makes it available to researchers. More details can be found in "The National COVID Cohort Collaborative (N3C): Rationale, design, infrastructure, and deployment" (https://academic.oup.com/jamia/article/28/3/427/5893482) . All data is available in the N3C Data Enclave to those with an approved protocol and data use request from an institutional review board. Data access is governed under the authority of the National Institutes of Health; more information on accessing the data can be found at https://covid.cd2h.org/for-researchers. See "The National COVID Cohort Collaborative (N3C): Rationale, design, infrastructure, and deployment" for additional detail on how data is ingested, managed, and protected within the N3C Data Enclave.

Human research participants

Policy information about studies involving human research participants and Sex and Gender in Research.

Reporting on sex and gender

All data is provided by contributing partner sites and standardized into the OMOP data model. The "Gender" domain in OMOP denotes the person's biological sex (details at https://www.ohdsi.org/web/wiki/doku.php?id=documentation:vocabulary:gender). Gender is not captured as a standard concept in OMOP and is not used in our study. The methodology for determining the sex of an individual is particular to the partner site and not reported in our source data.

Sex is accounted for in our study in both the inverse probability of treatment weighting as well as a covariate in all primary models. We have two cohorts. The first has 128,269 females and 70,245 males. The second has 30,819 females and 16,585 males. The imbalance is due at least in part to our inclusion criteria, which requires healthcare utilization prior to COVID-19 onset that is more likely among females.

Population characteristics

Both cohorts are made up of individuals with a recorded COVID-19 infection. They are diverse in age, race, and comorbidities. Out of 198,514 total individuals, 10.4% were 18-24, 18.4% were 25-34, 27.0% were 35-49, 25.5% were 50-64, and 18.7% were 65+ years old. The mean age was 47 years old. Additionally, 1.3% were Asian non-Hispanic, 13.3% were Black or African American non-Hispanic, 9.4% were Hispanic or Latino (any race), 0.1% were Pacific Islander non-Hispanic, 71.9% were White Non-Hispanic, 2.1% were other Non-Hispanic, and 1.8% were unknown.

Recruitment

Our study is a retrospective electronic health record (EHR) study filtered for individuals with a COVID-19 infection. Individuals from 75 medical centers comprising nearly 300 sites were available for inclusion.

Ethics oversight

The N3C data transfer to the United States National Center for Advancing Translational Sciences (NCATS) was approved under a Johns Hopkins University Reliance Protocol #IRB00249128 or individual site agreements with NIH. The use of human data for this study was approved by the Johns Hopkins Medicine Institutional Review Board (IRB) #IRB00279988 through a data use agreement entitled “Characterization of long-COVID: definition, stratification, and multi-modal analysis”. The N3C Data Enclave is managed under the authority of the NIH; information can be found at https://ncats.nih.gov/n3c/resources.

Note that full information on the approval of the study protocol must also be provided in the manuscript.

Field-specific reporting

Please select the one below that is the best fit for your research. If you are not sure, read the appropriate sections before making your selection.

☒ Life sciences ☐ Behavioural & social sciences ☐ Ecological, evolutionary & environmental sciences

For a reference copy of the document with all sections, see nature.com/documents/nr-reporting-summary-flat.pdf

Life sciences study design

All studies must disclose on these points even when the disclosure is negative.

Sample size

There was no predetermined sample size, all available individuals meeting the inclusion criteria were used in our retrospective EHR analysis. Our cohorts of 47,404 and 198,514 individuals are more than sufficient for the statistical models used, none of which had more than 45 degrees of freedom.

Data exclusions

Our study included the following pre-established inclusion criteria:
1. Having an ICD-10 COVID-19 diagnosis code or a positive SARS-CoV-2 PCR or antigen test between August 1, 2021 and January 31, 2022.
2. Having a recorded health care visit between 120 and 300 days after their COVID-19 indication.
3. Having at least two health care visits in the year prior their COVID-19 indication.

	4. Being at least 18 years old at the time of their COVID-19 indication.<br>5. Having either completed or not started a COVID-19 vaccine regimen at the time of their COVID-19 indication.<br><br>The rationale for the study period was to ensure that (1) long COVID diagnoses were commonplace no more than two months after COVID infection and (2) at least four months of post-COVID-19 history was available for every patient. The rationale for the second and third criteria was to ensure that those included in the study had established care at the facility contributing their data, decreasing the likelihood of the outcome going undiagnosed in our EHR. The rationale for the fourth and fifth criteria was our interest in the association between vaccination and long COVID diagnosis in adults. We considered those with incomplete vaccination regimen neither to be unvaccinated nor having the full potential protection of a completed regimen.<br><br>An additional pre-established exclusion criteria applied to the cohort using clinical diagnoses of long COVID. If the clinical diagnosis occurred within 45 days of their acute COVID-19 diagnosis date, then they were excluded. This is because the widely-used definitions of long COVID typically require symptoms to persist more than 45 days after the acute COVID-19 event to be considered long COVID. Therefore, the excluded patients either have an incorrect acute COVID-19 index date or a misaligned long COVID diagnosis; in either case they should be excluded.
Replication	All programming codes and value sets are publicly shared and available through N3C. Credentialed investigators or validated citizen scientists can onboard to N3C after appropriate human subjects training and Data Use Agreements to reproduce the results with the same data. Validation on a different population will be enabled through our open access code.<br><br>The reproducibility of the findings were validated through bootstrap samples of the available population and through sensitivity analyses. The bootstrap samples showed all findings to be robust to random variation resulting from different (but similarly sized) samples. Sensitivity analyses showed the findings to be robust to several factors that we considered most likely to influence reproducibility: methodological decisions on how to best account for population characteristics, the unreliability of vaccination data in electronic health records, and the inconsistency of available follow-up. A sensitivity analysis that evaluated robustness to computable phenotype threshold showed that the model-based analysis may not be reproducible using another threshold or a different computable phenotype.
Randomization	Our study was observational and retrospective; randomization was not possible. Covariates were controlled using inverse probability of treatment weighting (IPTW) and validated by evaluating covariate standardized mean differences between the exposed and unexposed groups. No absolute standardized mean differences were above 0.1 in either cohort after IPTW-adjustment. Evaluated covariates included age, race, sex, medical history, time of COVID-19 infection, and data partner site.
Blinding	Blinding was not relevant to our study due to its retrospective, observational design. Administration of the exposure was not included as a part of this study.

Reporting for specific materials, systems and methods

We require information from authors about some types of materials, experimental systems and methods used in many studies. Here, indicate whether each material, system or method listed is relevant to your study. If you are not sure if a list item applies to your research, read the appropriate section before selecting a response.

Materials & experimental systems

n/a	Involved in the study
☒	☐ Antibodies
☒	☐ Eukaryotic cell lines
☒	☐ Palaeontology and archaeology
☒	☐ Animals and other organisms
☒	☐ Clinical data
☒	☐ Dual use research of concern

Methods

n/a	Involved in the study
☒	☐ ChIP-seq
☒	☐ Flow cytometry
☒	☐ MRI-based neuroimaging
